# Supplementary material for: Progenitor translatome changes coordinated by Tsc1 increase perception of Wnt signals to end nephrogenesis
Source: Nat Commun. 2021 Nov 3;12:6332. doi: 10.1038/s41467-021-26626-9 (PMC8566581; doi:10.1038/s41467-021-26626-9)
Supplement: Supplementary file 1 — Supplementary information. [file 41467_2021_26626_MOESM1_ESM.pdf]

Supplementary Information

Progenitor Translatome Changes Coordinated by *Tsc1* Increase Perception of Wnt Signals to End Nephrogenesis

---

Alison E. Jarmas[1,2ϕ], Eric W. Brunskill[1,2ϕ], Praneet Chaturvedi[1,2], Nathan Salomonis[3], Raphael Kopan[1,2,*]

[1] Department of Pediatrics, University of Cincinnati College of Medicine, Cincinnati, OH, USA.

[2] Division of Developmental Biology, Cincinnati Children's Hospital Medical Center, Cincinnati, OH, USA

[3] Division of Biomedical Informatics, Cincinnati Children's Hospital Medical Center, Cincinnati, OH, USA

*Author for correspondence: Raphael.Kopan@cchmc.org

ϕ These authors contributed equally to the manuscript

Supplementary Figure 1

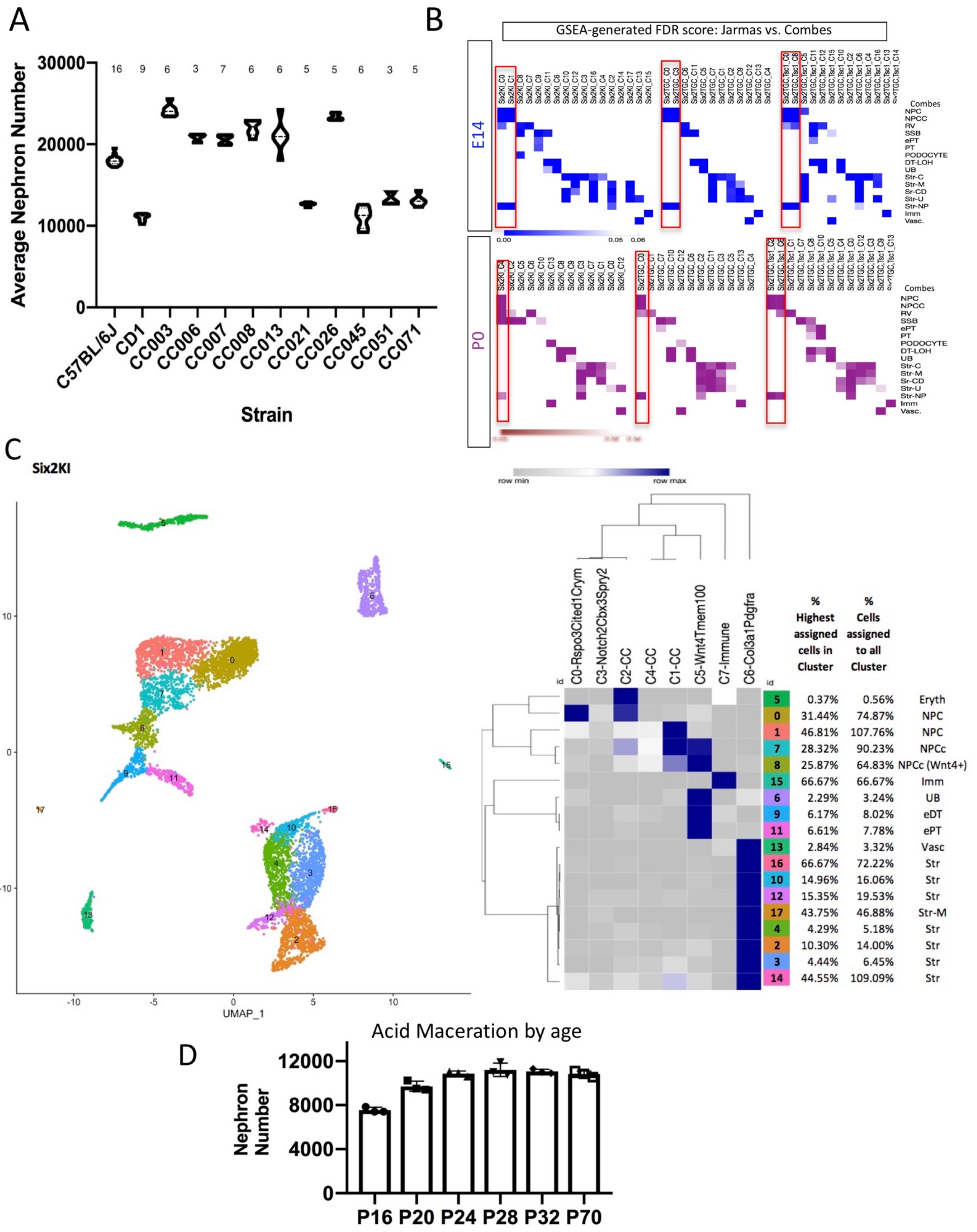

Supplementary Figure 1 (continued)

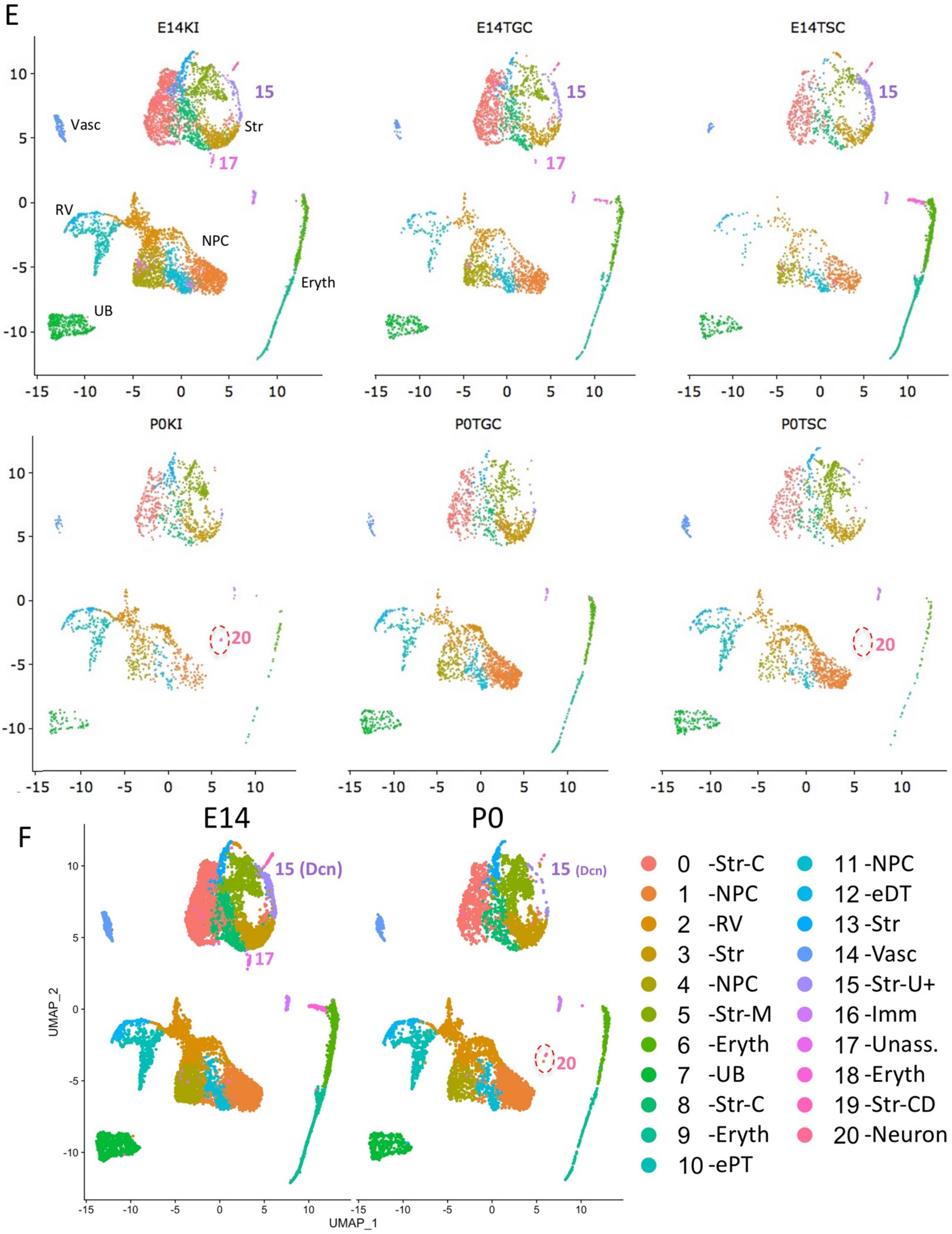

Supplementary Figure 1 (continued)

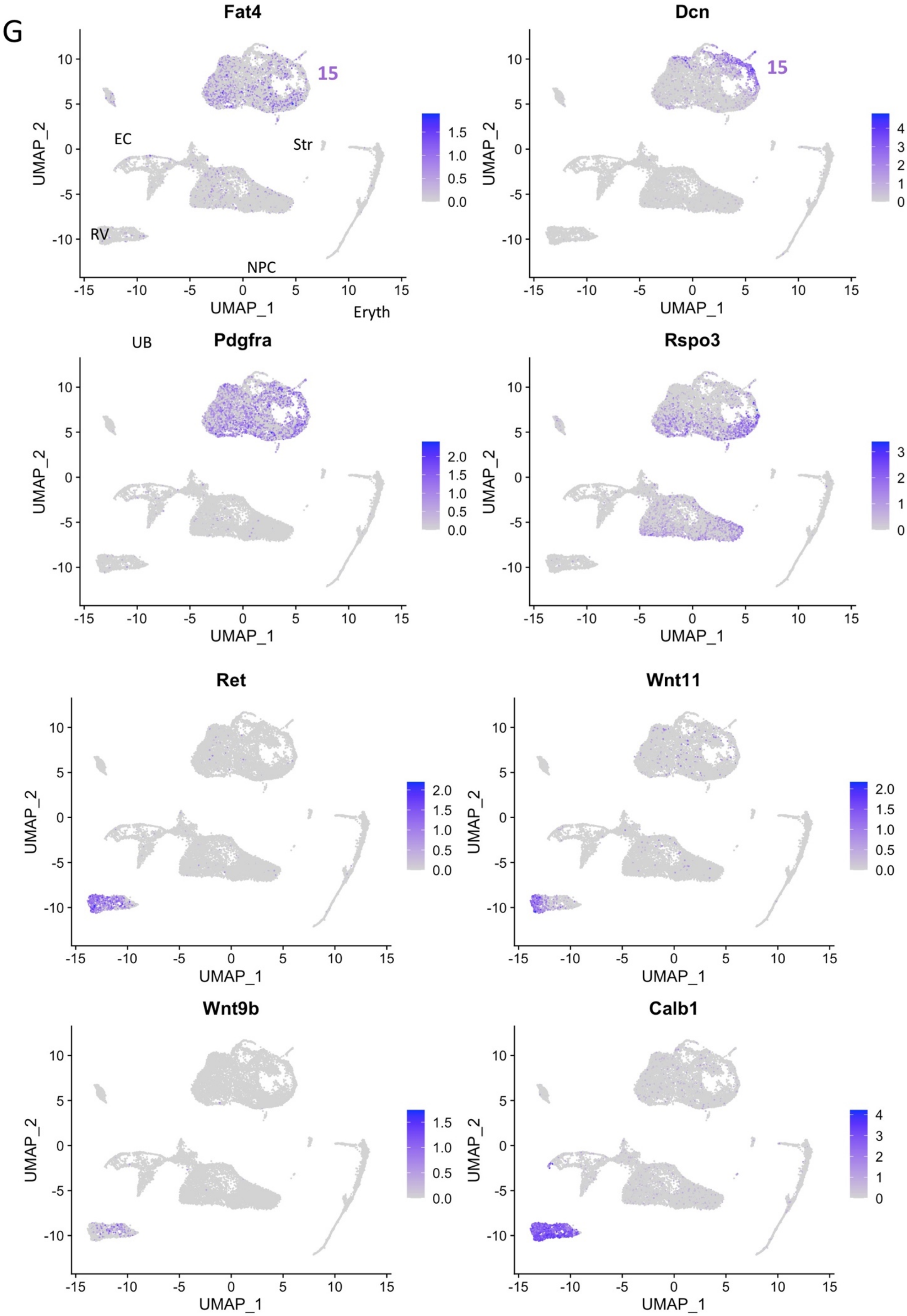

Supplementary Figure 1 (continued)

H

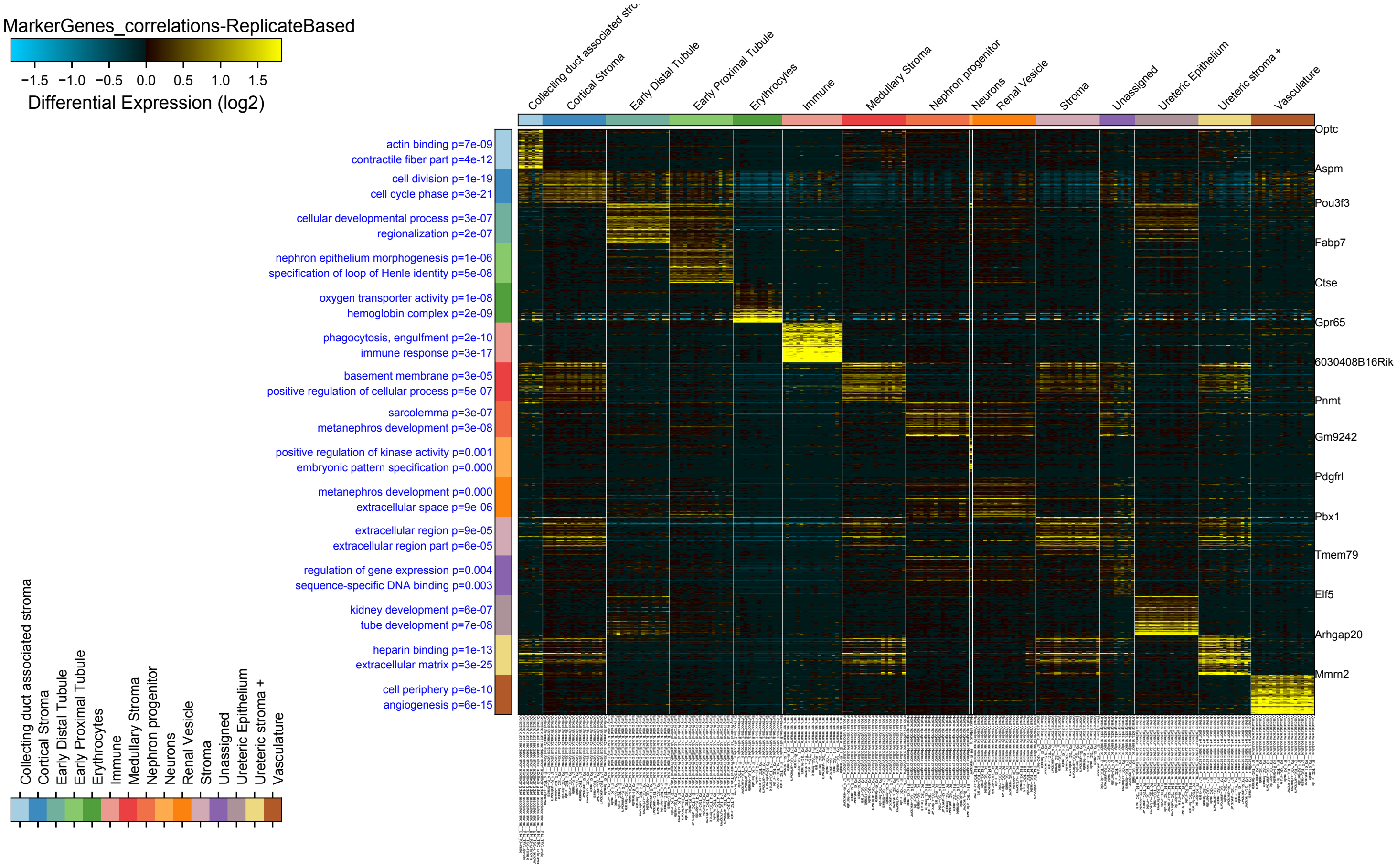

Supplementary Figure 1 (continued)

l

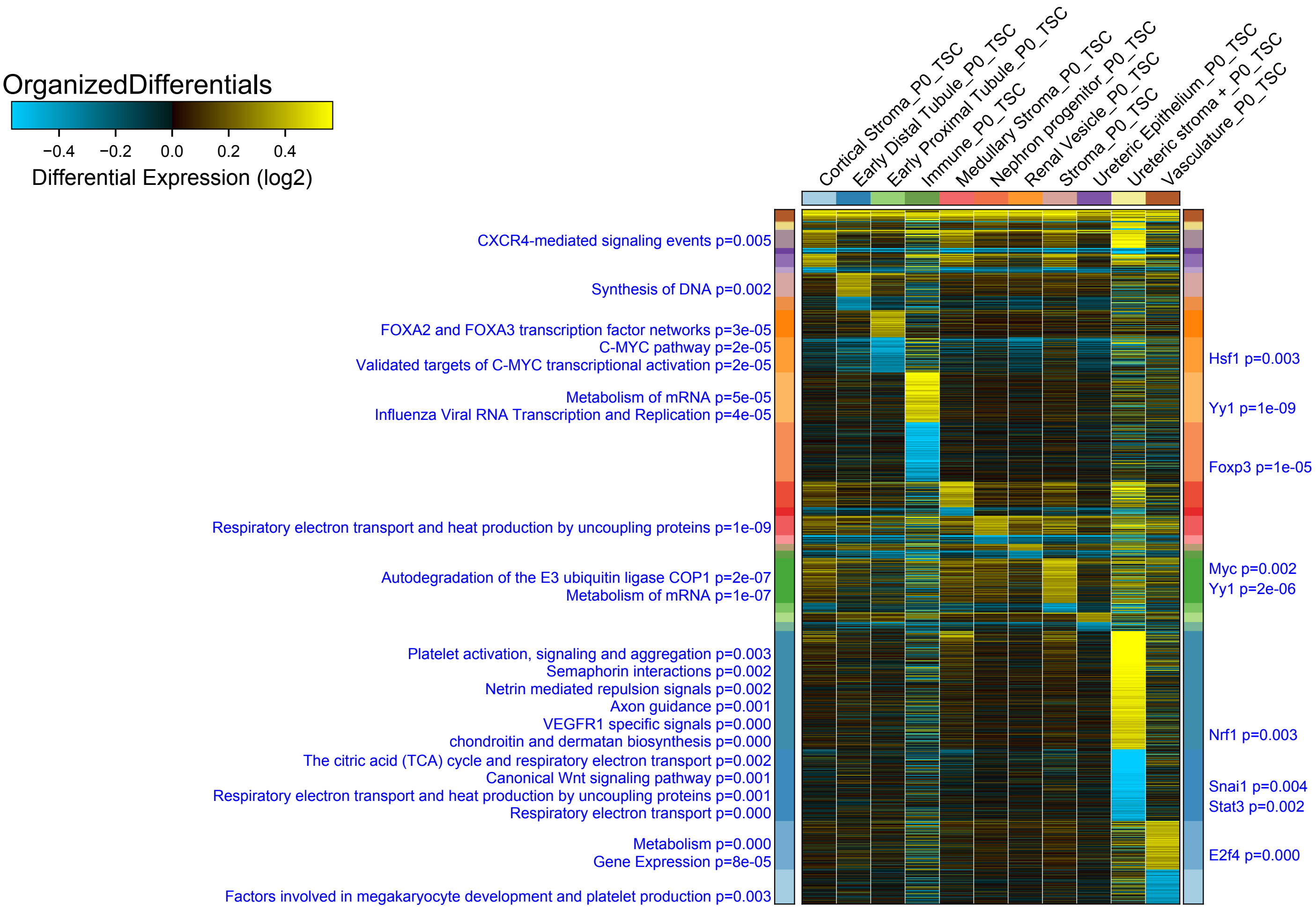

Supplementary Figure S1: Nephron number, integration analyses across strains and GSEA analysis of scRNA-seq data. (A) Violin plots of nephron number (NN) variation among individuals of the same strain. Each data point represents the average nephron count of the two kidneys per animal. The number of animals tested is listed above each strain. Note the variance within the inbred C57BL/6J is not smaller than that seen in eight-parent strains from the Collaborative Cross (CC strains) or outbred CD1. Note also that NN cluster along a high value (C57BL/6J, CC003, CC006, CC007, CC008, CC013, CC021, CC026) or a low value (CD1, CC021, CC045, CC051, CC071) independent of body weight (CD1 mice are larger than C57BL/6J mice) NN were counted by acid maceration in animals older than P28 (after P24 glomeruli are resistant to acid, demonstrated in (D) for nephron counts of CD1 mice with n = 3 animals per group; error bars represent standard deviation from the mean). (B) GSEA analysis compared each of the clusters identified (as in Figure 1A) with the clusters identified by Combes et al. Best FDR value for each two-way comparison was extracted with a custom script (available in GitHub) to generate the similarity matrix shown. Note that only Cluster C5 in one of six samples (E14 $Six2^{TGC; Tsc1}$) contained both stromal and NPC markers and was excluded from downstream analyses. Clusters in the red boxes were used for the NPC analyses shown in the text. (C) UMAP of E14 $Six2^{KI}$ cortically enriched kidney cells from 3 pooled embryos established 14 major clusters and 4 minor ones. 6 clusters formed the NPC "continent", and 7 formed the stromal continent. Nearest Neighbor based correction assigned cells to the NPC clusters seen in Combes et al. All cells in NPC cluster 1 were correlated with Combes, ~47% with C1-CC. 31% of NPC cluster 0 correlated with Combes C0, the rest with C2-CC. Similar results were obtained with GSEA, establishing correlation with FDR score <0.05 (B). "% Highest assigned cells in cluster" shows how many cells in our clusters were assigned to an individual Combes cluster. "% Cells assigned to all clusters" shows how many cells in our clusters were assigned to any of the Combes cluster (Note that cluster 14 cells were assigned to multiple Combes clusters, resulting in 109% assigned cells). (E) The contribution of each sample to the Seurat integration UMAP presented in Figure 1C is shown. Note the consistent contributions to the cluster architecture across age and genotype. Cluster 17 (unassigned), 15 (Str), and 20 (neurons) are the only clusters not present in every sample. (F) Seurat Integration UMAP presented for pooled E14 and P0 samples. Note cluster 15, characterized by *Dcn* expression, is more prominent in the younger samples. (G) Selected feature plots identifying the stromal continent (*Pdgfra*) and UB/tips cells (*Calb1, Ret, Wnt9b*). NPC and stromal expression of *Rspo3* are identified. (H) MarkerFinder analysis (AltAnalyze) of all cell types assigned male, female or unassigned reveals consistent gene expression patterns across these biological pseudo-replicates. Each column on lower x-axis represents a unique cell type, age/genotype and assigned sex combination. (I) Differential expression of $Six2^{TGC; Tsc1}$ compared to control at P0 across cell types identifies enrichment in respiratory electron transport in NPCs; significance was determined using an unadjusted two-tailed t-test with p <0.05 as significant.

Supplementary Figure 2

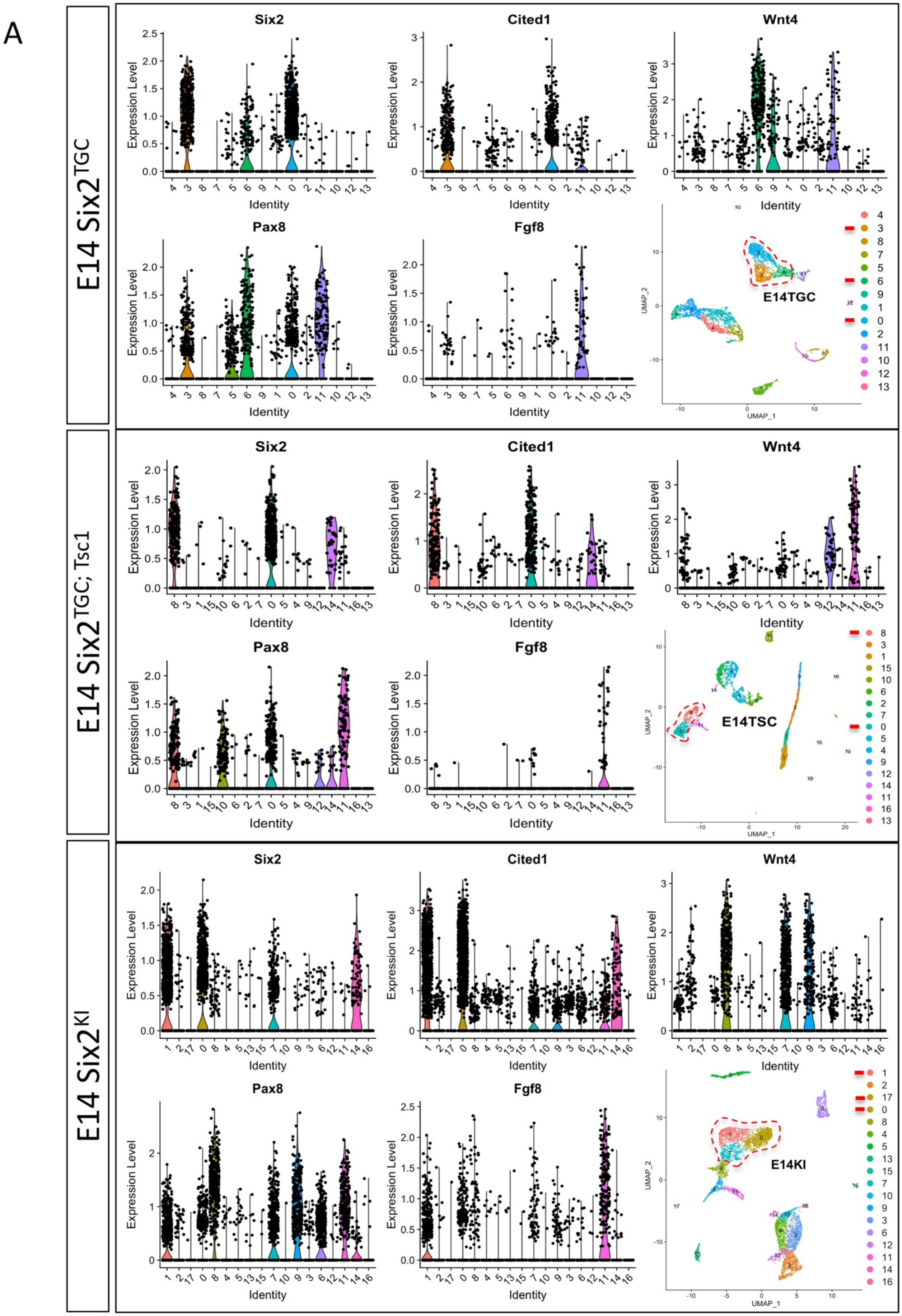

Supplementary Figure 2 (continued)

B

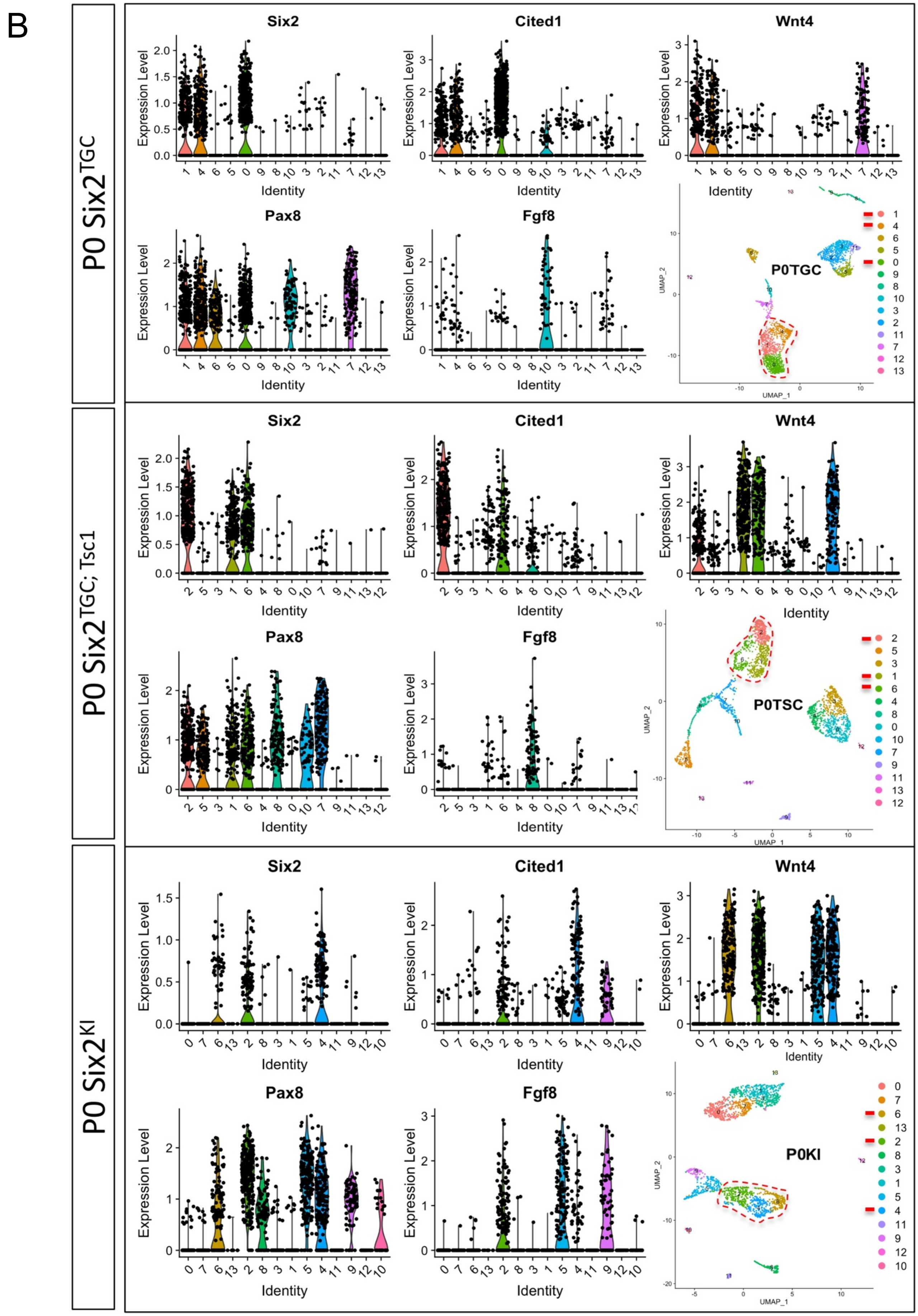

Supplementary Figure S2: Violin plots for selected markers reflecting nephron progenitor commitment. Gene expression of E14 (A) and P0 (B) of Six2^{TGC} (top row), $\text{Six2}^{\text{TGC; Tsc1}}$ (middle) and Six2^{KI} (bottom row). UMAP diagram added to identify clusters (NPC encircled in a red, dashed line). Gene name shown above the plots. The clusters in plot are ordered from left (top in the UMAP) to right (bottom in UMAP, enlarge to view). NPC clusters are also marked with red box near the cluster numbers in the UMAP. Note that at E14, *Six2+; Cited1+* (*Wnt4–*) clusters are present in both genotypes, and the same is true for C0 in P0 Six2^{TGC}. By contrast, all NPC clusters in $\text{Six2}^{\text{TGC; Tsc1}}$ at P0 are *Six2+; Cited1+*; *Wnt4+* though C2 has the least amount of Wnt4.

Supplementary Figure 3

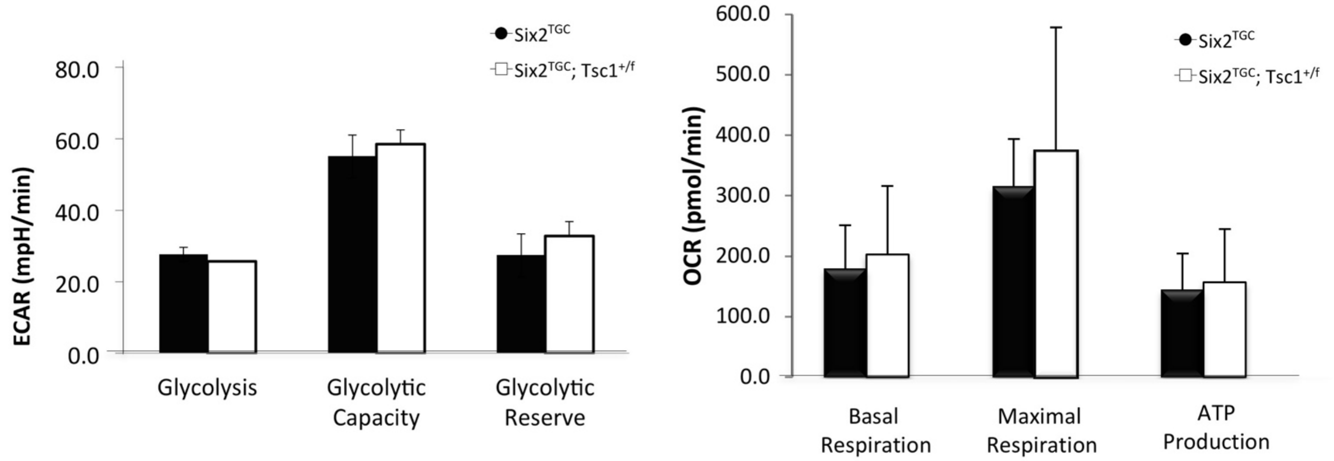

Supplementary Figure S3: Quantification of *in vitro* metabolic assays in nephron progenitor cells. Seahorse glycolysis stress test and cell mito stress test assays reveal no statistical difference in glycolytic/respiration parameters in NPCs isolated at P0 (controls and $Tsc1^{+/-}$) that were cultured for a week. Data is aggregated from 2-3 replicate wells per animal and multiple animals per genotype (n = 3 control $Six2^{TGC}$ and n = 3 $Six2^{TGC;\ Tsc1}$ animals for glycolysis and n = 3 control $Six2^{TGC}$ and n = 4 $Six2^{TGC;\ Tsc1}$ animals for mitochondrial respiration). Error bars represent standard deviation above the mean parameter values. Source data are provided as a Source Data file.

Supplementary Figure 4

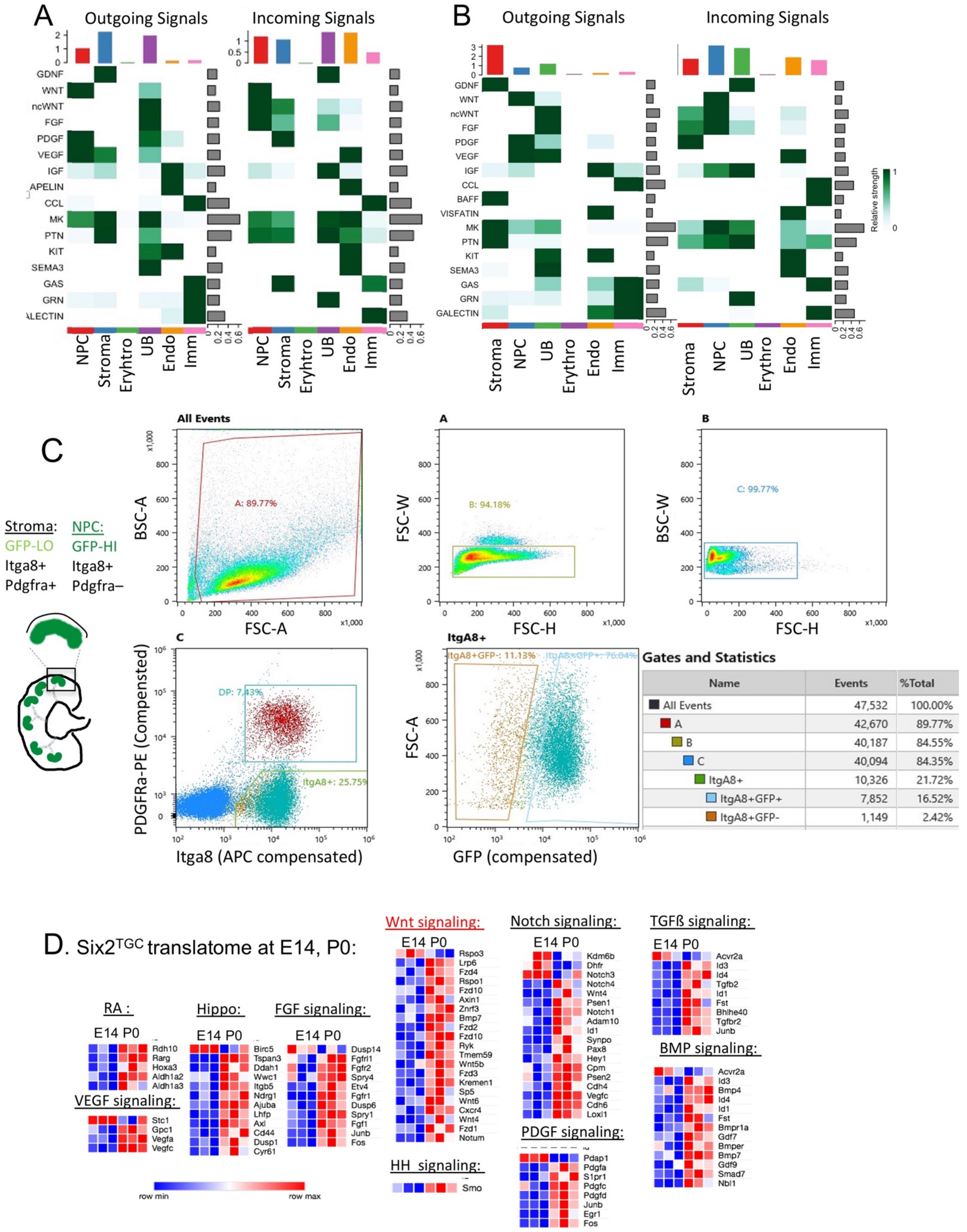

Supplementary Figure 4 (continued)

E

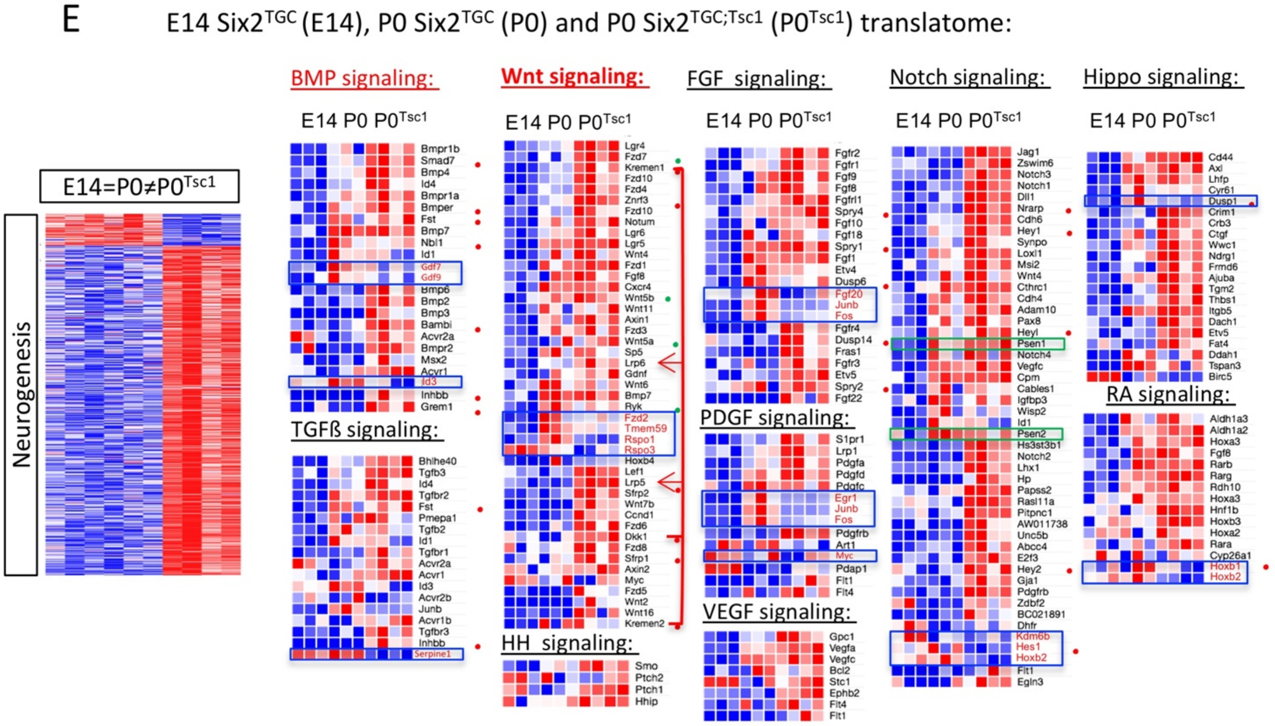

F

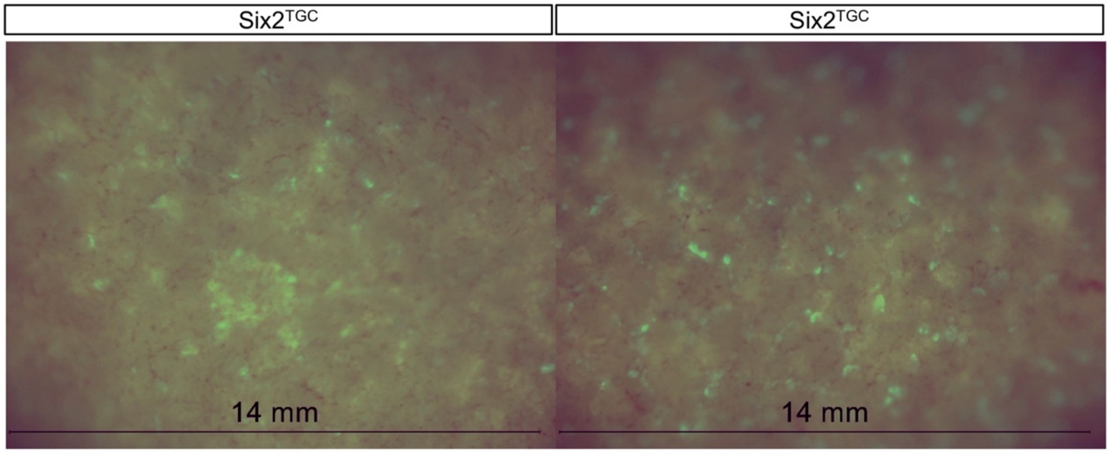

Supplementary Figure S4: Analysis of signaling pathway components by single-cell transcriptome and TRAP. (A-B) Summary of CellChat analysis incoming and outgoing signaling patterns inferred from scRNA-Seq samples pooled by age (E14 in (A), P0 in (B)) and then analyzed by cell type does not identify any robust changes in signaling networks correlated with NPC aging based on transcription. (C) Schematic of sorting strategy (used for isolation of NPCs for data presented in Figures 3B, 3C, 4A, 4B, 4B', 4C, and Supplementary Figures S3, S4D, and S4E) depicts the Itga8+, Pdgfra-, GFP^{HI} NPC population with the compensation and gating strategy shown. The PDGFRa antibody is conjugated to PE; the biotinylated ITGA8 antibody is detected by streptavidin-APC. (D) RNAseq analysis of polysomal transcripts is shown in Supplementary Data 9. We selected transcripts based on membership in ten signaling pathways (appearing first in the table). The data were filtered to only show avg expression of Log2≥1; fold change >1.2 or <-1.2, and raw pValue <0.05 in at least one of the datasets in the comparison. Transcripts that vary between E14 $Six2^{TGC}$ and P0 $Six2^{TGC}$, visualized to show high/low values within each row. (E) Transcripts unique to P0 $Six2^{TGC; Tsc1}$ reflect enrichment for neurogenesis. Signaling transcripts that vary between E14 $Six2^{TGC}$, P0 $Six2^{TGC}$ and P0 $Six2^{TGC; Tsc1}$, visualized to show high/low values within each row. Antagonists are marked by a red dot, non-canonical components marked by green dot. Transcripts differentially translated in *Tsc1* hemizygotes are enclosed in a blue box and marked with red text. DKK1 and Kremen protein target Lrp4/5/6 for degradation, shown with red lines and arrows. Note that the catalytic subunit in g-secretase, required to produce Notch signals (Psen1, Psen2), are expressed poorly in E14 (green boxes). (F) Visualization of GFP in additional P4 littermates from Figure 4D; this experiment was repeated for 3 litters with similar results obtained in each case.

Supplementary Figure 5

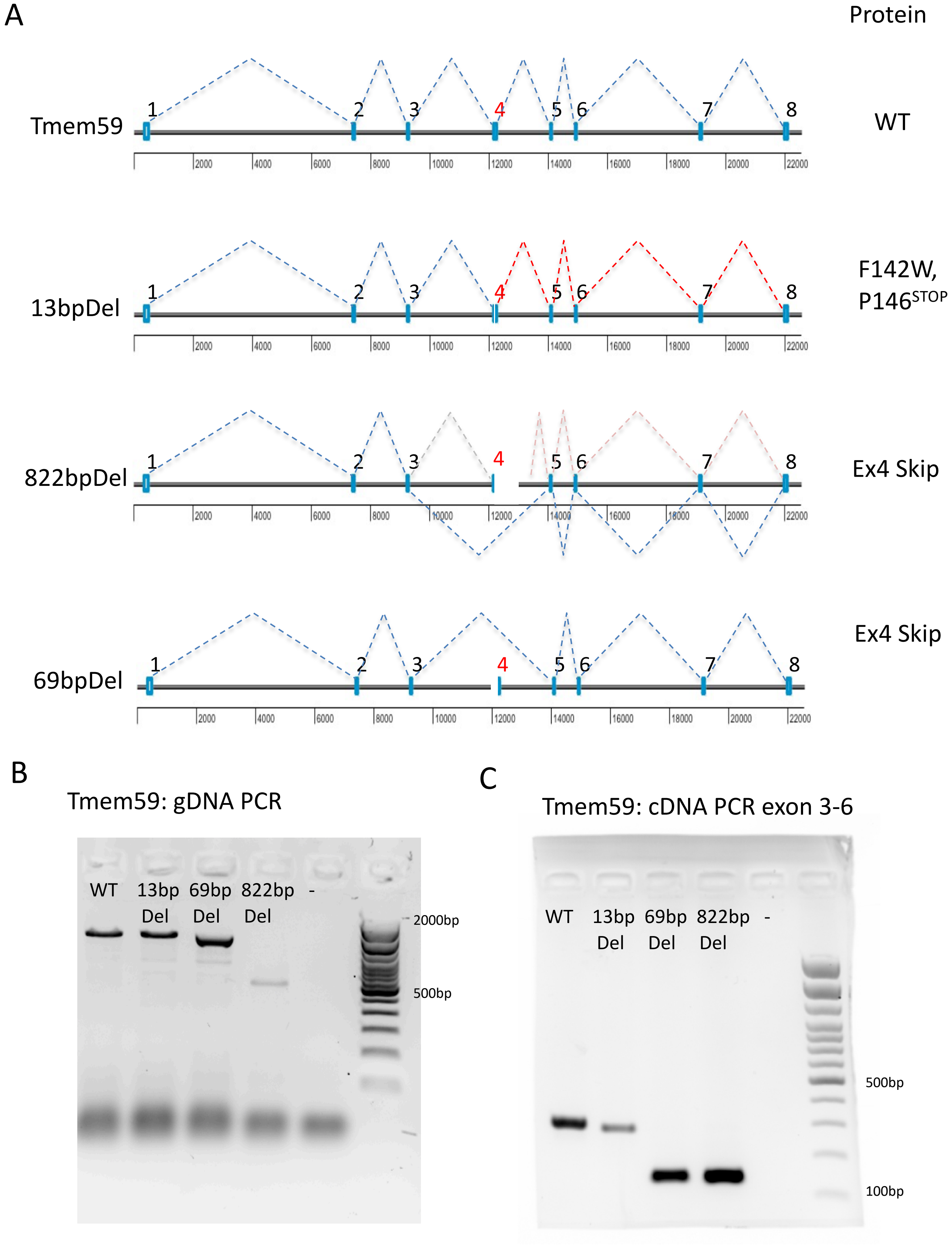

Supplementary Figure 5 (continued)

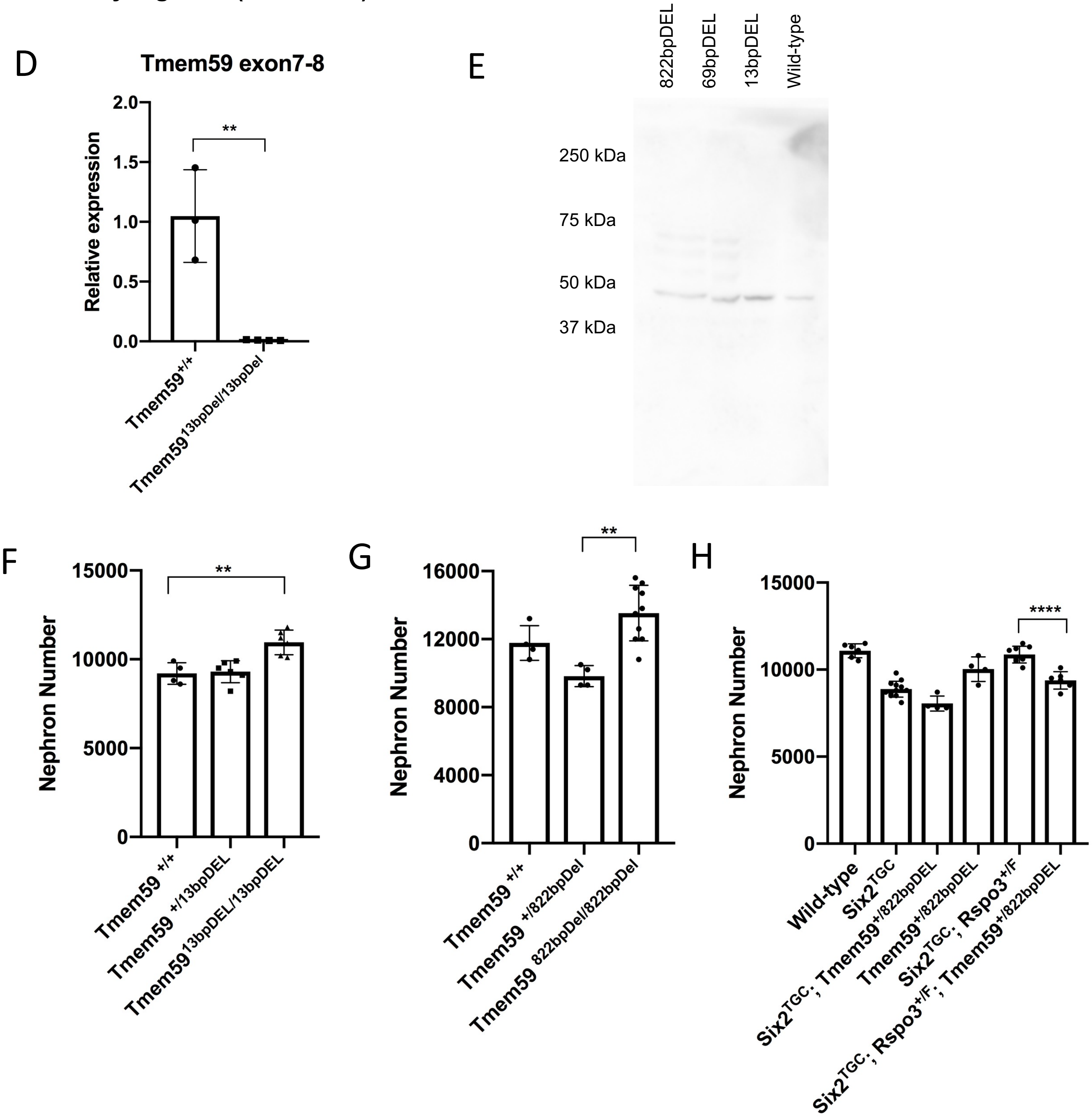

Supplementary Figure S5: Characterization and analysis of *Tmem59* mutant mice. (A) Schematic of wild-type and mutant (13bpDEL, 822bpDEL and 69bpDEL) alleles used in this study with protein product predicted from cDNA sequencing of the PCR product shown in ((C); see also Supplementary Data 10). (B) Genomic PCR identifying *Tmem59* alleles used in this study. For the genomic DNA (B) and cDNA (C) gels, consistent results were obtained for all experimental animals included in this manuscript. (D) RT-qPCR for the c-terminal end of *Tmem59* reveals a loss of mRNA transcripts in homozygous *Tmem59*$^{13bpDEL/13bpDEL}$ mutants. Error bars represent standard deviation from the sample group mean. Two-tailed unpaired t tests were performed in GraphPad Prism version 8 to evaluate statistical significance of Tmem59 expression data. ** denotes $p \leq 0.01$ ($p = 0.0026$) (E) Representative western blot with ThermoFisher PA5-21575 antibody failed to identify TMEM59 band at predicted molecular weight and does not show a truncated protein product; similar results were obtained with three antibodies tested (listed in Methods). (F-H) Nephron counts in adult ($\geq$ P28) mice determined via the acid maceration method for glomerular counts. One-way ANOVA with Tukey's multiple comparisons tests were performed in GraphPad Prism version 8 to evaluate statistical significance of single kidney nephron numbers. **

denotes $p \leq 0.01$, **** denotes $p \leq 0.0001$. (F) $p = 0.0027$ (G) $p = 0.0010$ and (H) $p \leq 0.0001$. Both kidneys were counted for each animal with males and females represented. Error bars represent standard deviation from the sample group mean. Source data are provided as a Source Data file.

Supplementary Table 1. Primer Sequences (5’ – 3’) used in this study.

Primer Name	Primer Sequence
Cre genotyping – F	GCATTACCGGTCGATGCAACGAGTGATGAG
Cre genotyping – R	GAGTGAACGAACCTGGTCGAAATCAGTGCG
Tsc1 genotyping – F	AGGAGGCCTCTTCTGCTACC
Tsc1 genotyping – R	CAGCTCCGACCATGAAGTG
Mtor genotyping – F	TTATGTTTGATAATTGCAGTTTTGGCTA
Mtor genotyping – R	TTTAGGACTCCTTCTGTGACATACATTTC
Rspo3 genotyping – F	TATACTGCGATCTAATGCCCTCT
Rspo3 genotyping – R	CCTTTCGACTTGATGGTGG
Tmem59 genotyping – F	AAGTTATCTGTAACCTCTTCCA
Tmem59 genotyping – R1	GTCCTGCTGCTCGGGTTTC
Tmem59 genotyping – R2	TGCACAGCTTCCTCATCAAC
Tmem59-exon7-8 – F qPCR	TTGCTCTGGATCTGTTGTG
Tmem59-exon7-8 – R qPCR	AGGAGCTGGGTATCTGCTCA
Tmem59-exon3-6 – F qPCR	TTCCCAACCTGATGAGCAGT
Tmem59-exon3-6 – R qPCR	AGCCATCGCTTTCTTCCTCT
Rspo3 qPCR – F	TTGACAGTTGCCCAGAAGGG
Rspo3 qPCR – R	CTGGCCTCACAGTGTACAATACT
Gapdh qPCR – F	AGGTCGGTGTGAACGGATTTG
Gapdh qPCR – R	TGTAGACCATGTAGTTGAGGTCA
Lgr5 qPCR – F	CCTGTCCAGGCTTTCAGAAG
Lgr5 qPCR – R	CTGTGGAGTCCATCAAAGCA
Fzd10 qPCR – F	TATGAACGCCTCAACATGGA
Fzd10 qPCR – R	TCAGGCAGTCAGGTGTCTTG
