# Supplementary material for: Progenitor translatome changes coordinated by Tsc1 increase perception of Wnt signals to end nephrogenesis
Source: Nat Commun. 2021 Nov 3;12:6332. doi: 10.1038/s41467-021-26626-9 (PMC8566581; doi:10.1038/s41467-021-26626-9)
Supplement: Supplementary file 3 — Reporting summary. [file 41467_2021_26626_MOESM3_ESM.pdf]

nature research

Corresponding author(s): Raphael Kopan

Last updated by author(s): Sep 24, 2021

Reporting Summary

Nature Research wishes to improve the reproducibility of the work that we publish. This form provides structure for consistency and transparency in reporting. For further information on Nature Research policies, see our Editorial Policies and the Editorial Policy Checklist.

Statistics

For all statistical analyses, confirm that the following items are present in the figure legend, table legend, main text, or Methods section.

n/a	Confirmed	
☐	☒	The exact sample size (*n*) for each experimental group/condition, given as a discrete number and unit of measurement
☐	☒	A statement on whether measurements were taken from distinct samples or whether the same sample was measured repeatedly
☐	☒	The statistical test(s) used AND whether they are one- or two-sided<br>*Only common tests should be described solely by name; describe more complex techniques in the Methods section.*
☒	☐	A description of all covariates tested
☐	☒	A description of any assumptions or corrections, such as tests of normality and adjustment for multiple comparisons
☐	☒	A full description of the statistical parameters including central tendency (e.g. means) or other basic estimates (e.g. regression coefficient) AND variation (e.g. standard deviation) or associated estimates of uncertainty (e.g. confidence intervals)
☐	☒	For null hypothesis testing, the test statistic (e.g. *F*, *t*, *r*) with confidence intervals, effect sizes, degrees of freedom and *P* value noted<br>*Give P values as exact values whenever suitable.*
☒	☐	For Bayesian analysis, information on the choice of priors and Markov chain Monte Carlo settings
☒	☐	For hierarchical and complex designs, identification of the appropriate level for tests and full reporting of outcomes
☒	☐	Estimates of effect sizes (e.g. Cohen's *d*, Pearson's *r*), indicating how they were calculated

Our web collection on statistics for biologists contains articles on many of the points above.

Software and code

Policy information about availability of computer code

Data collection	Agilent Technologies Seahorse Report Generator (v3.0.8 and v4.03) was used in metabolic data collection.
Data analysis	Raw sequencing data of all samples were processed using the CellRanger workflow (version 2.0.0). Further analysis was completed with the R packages Seurat (versions 2.3.4 and 3.2.3) and CellChat (v 1.0.0). For scRNA-seq and bulk RNA-seq data, AltAnalyze (v2.14) using Kallisto was used (http://altanalyze.org). All computer codes and scripts [R scripts, custom Python/Perl scripts and software packages] have been uploaded to GitHub [https://github.com/praneet1988/SingleCellAnalysisScripts]. All codes have been licensed with a general public license (GPL v3.0).<br><br>Other software used:<br>GraphPad Prism 8<br>Imaris 9.6.0<br>FIJI 2.1.0/1.53c<br>Aperture 3.6<br>Morpheus (version not available)

For manuscripts utilizing custom algorithms or software that are central to the research but not yet described in published literature, software must be made available to editors and reviewers. We strongly encourage code deposition in a community repository (e.g. GitHub). See the Nature Research guidelines for submitting code & software for further information.

Data

Policy information about availability of data

All manuscripts must include a data availability statement. This statement should provide the following information, where applicable:

- Accession codes, unique identifiers, or web links for publicly available datasets
- A list of figures that have associated raw data
- A description of any restrictions on data availability

The RNA-sequencing data generated in this study have been deposited in NCBI's Gene Expression Omnibus (GEO) (Edgar et al., 2002) database under accession codes GSE173264 (https://www.ncbi.nlm.nih.gov/geo/query/acc.cgi?acc=GSE173264), GSE173265 (https://www.ncbi.nlm.nih.gov/geo/query/acc.cgi?acc=GSE173265), and GSE173266 https://www.ncbi.nlm.nih.gov/geo/query/acc.cgi?acc=GSE173266), and contain all processed bam files, raw counts of genes across barcodes/cells and cell annotations in the form of a metafile. Source data are provided with this paper.

Field-specific reporting

Please select the one below that is the best fit for your research. If you are not sure, read the appropriate sections before making your selection.

- [x] Life sciences
- [ ] Behavioural & social sciences
- [ ] Ecological, evolutionary & environmental sciences

For a reference copy of the document with all sections, see nature.com/documents/nr-reporting-summary-flat.pdf

Life sciences study design

All studies must disclose on these points even when the disclosure is negative.

Sample size	Sample sizes were not calculated prior to experimentation and were determined from previous literature (Volovelsky et al, 2018; Cebrian et al, 2014) evaluating nephron number phenotypes. Sample sizes for cessation timing (postnatal day 4 surface imaging) experiments were limited to the litter size due to the need to control for differences in precise gestational timing.
Data exclusions	All data were included in the subsequent analysis, with the exception of single-cell RNA sequencing data: low-quality cells were filtered out as described in the Methods section, consistent with standard practices in the field.
Replication	Biological (n = 5 independent samples) and technical (n = 3) replicates were utilized to ensure reproducibility in RT-qPCR. Nephron count samples were quantified in triplicate with consistent results. Postnatal day 4 surface imaging to visualize GFP (delayed nephrogenesis cessation) was performed in 3 litters with similar findings.
Randomization	For pimonidazole injection experiments, a random littermate animal was selected as an uninjected control for image analysis. For nephron counts, all littermates were evaluated prior to genotyping. For experiments involving sorting of GFP+ NPCs, all GFP+ embryos or pups in a litter were used.
Blinding	All nephron number experiments were performed blind to the genotype of each sample. Postnatal day 4 surface images were obtained and evaluated prior to assessment of genotype. Flow cytometry analysis of total protein and quantification of pimonidazole staining and Axin2 transcripts by RNAScope were performed while blinded to the genotype of the sample. Single-cell RNA-Sequencing libraries were independently prepared blinded to the sample genotype. Blinding was not involved in RT-qPCR studies due to need for pooling of samples to generate replicates.

Reporting for specific materials, systems and methods

We require information from authors about some types of materials, experimental systems and methods used in many studies. Here, indicate whether each material, system or method listed is relevant to your study. If you are not sure if a list item applies to your research, read the appropriate section before selecting a response.

Materials & experimental systems

n/a	Involved in the study	
[ ]	[x]	Antibodies
[x]	[ ]	Eukaryotic cell lines
[x]	[ ]	Palaeontology and archaeology
[ ]	[x]	Animals and other organisms
[x]	[ ]	Human research participants
[x]	[ ]	Clinical data
[x]	[ ]	Dual use research of concern

Methods

n/a	Involved in the study	
[x]	[ ]	ChIP-seq
[ ]	[x]	Flow cytometry
[x]	[ ]	MRI-based neuroimaging

Antibodies

Antibodies used	Anti-GFP (Aves Labs, AB_2307313, Catalog #GFP-1020)<br>Anti-Phospho-S6 Ribosomal Protein (Ser240/244) (Cell Signaling Technology, (D68F8) XP® Rabbit mAb #5364)<br>Anti-Cytokeratin 8+ 18 (Abcam, ab194130, discontinued)<br>Anti-pimonidazole (Hypoxyprobe Mab-4.3.11.3, Lot #04-11-19)<br>Anti-mIntegrin a8 (R&D Systems, Catalog Number BAF4076, Lot #ZDK0117051 and ZDK0219021)<br>Anti Mo CD140a (PDGFRA) (Invitrogen, Clone: APA5, Lot # 2049418, Catalog # 12-1401-81)<br>Anti-Six2 (Proteintech, 11561-2-AP)<br>Anti-Frizzled 10 (Proteintech, 18175-1-AP)<br>Anti-Lgr5 (R&D Systems, Lgr5/GPR49, Catalog #MAB82401, Lot #CIRN021810A, Clone #889901)<br>Anti-Tmem59 (Invitrogen, PA5-21575, Lot # VG3035934B)<br>Anti-GFP (AB_2716736, Catalog #HtzGFP-19F7 and AB_2716737, Catalog #HtzGFP-19C8; Memorial Sloan-Kettering Monoclonal Antibody Facility)<br>anti-Rabbit Cy3 (Jackson Immuno Research, Catalog #711-165-152)<br>anti-Chicken FITC (Jackson Immuno Research, Catalog #703-095-155)<br>Alexa Flour 488 donkey anti-chick (Jackson Immuno Research, Catalog #703-545-155)<br>Alexa Flour 647 goat anti-guinea pig (ThermoFisher Scientific, Catalog #A-21450)<br>Donkey anti-Chick IgY Cy3 (Jackson Immuno Research, Caalog #703-165-155)
Validation	Anti-GFP (validated by Western blot and immunohistochemistry in transgenic mice expressing GFP)<br>Anti-Phospho-S6 Ribosomal Protein (validated for WB, IHC, IF and flow cytometry in mouse tissue and cell extracts)<br>Anti-Cytokeratin 8+ 18 (discontinued; validated for western blot and immunohistochemistry across multiple species and tissue sources)<br>Anti-pimonidazole (per manufacturer, MAb1 is a mouse IgG1 monoclonal antibody (MAb) clone 4.3.11.3 supplied as a filter-sterilized, exhausted supernatant from hybridoma clone 4.3.11.3)<br>Anti-mIntegrin a8 (validated for mouse and rat via WB, IHC and flow cytometry)<br>Anti Mo CD140a (PDGFRA) (validated in NIH/3T3 cells via flow cytometry)<br>Anti-Six2 (validated for human, mouse and rat via Western blot and IF)<br>Anti-Frizzled 10 (validated in WB, IHC, IF,ELISA applications and shows reactivity with human, mouse, rat samples)<br>Anti-Lgr5 (validated in HEK cells transfected with mouse Lgr5 by flow cytometry)<br>Anti-Tmem59 (validated by Western blot for mouse tissue lysates with multiple unidentified bands present; of note, this manuscript reports failure to detect wild-type protein or mutation/truncated product)<br>Anti-GFP (AB_2716736, Catalog #HtzGFP-19F7 and AB_2716737, Catalog #HtzGFP-19C8): Nature Protocols 9, 1282–1291 (2014)

Animals and other organisms

Policy information about studies involving animals; ARRIVE guidelines recommended for reporting animal research

Laboratory animals	Laboratory animals used in this study are of the species Mus musculus on a mixed genetic background. The following strains were used: Tg(Six2-EGFP/cre)1Amc (Jax Stock No: 009600; herein Six2TGC), Six2-CreERT2, (herein Six2KI) 19, Tsc1f/f 77, B6 ;129S4 -Gt (ROSA)26Sortm9(EGFP/Rpl10a)Amc/J (Jax Stock No: 024750; herein EGFP/Rpl10a), Mtorf/f;78, Rspo3f/f (Jax Stock No: 027313; Rspo3tm1.1Jcob), Cited1-CreERT2-GFP+/tg; 20 and Foxd1tm1(GFP/cre)Amc (herein Foxd1Cre; Jax Stock No: 012463). Further, the following defined Collaborative Cross strains were used (CC003, CC006, CC007, CC008, CC013, CC021, CC026, CC045, CC051, and CC071). Both male and female animals were used for adult kidney analysis at age postnatal day 28. Embryos (embryonic day 14; E14) used for sequencing studies were pooled and sex was not determined.
Wild animals	No wild animals were used in the study.
Field-collected samples	No field collected samples were used in the study.
Ethics oversight	All animal study protocols were approved and supervised by the Cincinnati Children's Hospital Medical Center Institutional Animal Care and Use Committee (IACUC2018-0107, IACUC2018-0108, IACUC2016-0022, IACUC2016-0032)

Note that full information on the approval of the study protocol must also be provided in the manuscript.

Flow Cytometry

Plots

Confirm that:

- [x] The axis labels state the marker and fluorochrome used (e.g. CD4-FITC).
- [x] The axis scales are clearly visible. Include numbers along axes only for bottom left plot of group (a 'group' is an analysis of identical markers).
- [x] All plots are contour plots with outliers or pseudocolor plots.
- [x] A numerical value for number of cells or percentage (with statistics) is provided.

Methodology

Sample preparation	Embryonic (E14) or early postnatal (P0, P1, P2) mouse kidneys expressing GFP were isolated in cold PBS, digested with cold protease at 10 degrees C or neutral protease at 20 degrees C for 10 minutes, washed, and stained with Itga8 (biotinylated) and PDGFRA primary antibodies followed by streptavidin-APC labeling for FACS isolation. See methods for additional details.
Instrument	Sony SH800S, BD FACS Canto II
Software	FlowJo software was used to analyze flow cytometry data.
Cell population abundance	For cell sorting, post-sort cell pellets were visualized for GFP expression; sample purity was not routinely quantified.
Gating strategy	The gating strategy and division of positive/negative populations is visualized in Supplementary Figure 4C; FSC and BSC were used to exclude debris and doublets outside gates A, B and C. Panel C shows double positive (Itga8/Pdgfra, designated DP) cells as well as Itga8+ cells, the latter of which were then selected for GFP positivity. A GFP- kidney sample was used to establish the boundaries for GFP; unstained controls were similarly used for positive/negative gate definitions for Itga8 (APC) and PDGFRa (PE).

☒ Tick this box to confirm that a figure exemplifying the gating strategy is provided in the Supplementary Information.
