# Supplementary material for: Progenitor translatome changes coordinated by Tsc1 increase perception of Wnt signals to end nephrogenesis
Source: Nat Commun. 2021 Nov 3;12:6332. doi: 10.1038/s41467-021-26626-9 (PMC8566581; doi:10.1038/s41467-021-26626-9)
Supplement: Supplementary file 4 — Description of Additional Supplementary Files. [file 41467_2021_26626_MOESM4_ESM.pdf]

Description of Additional Supplementary Files

Supplementary Data 1. Clustering and top marker genes for E14 *Six2*^{KI} scRNA-Seq sample.

Supplementary Data 2. GO-Elite analysis of E14 *Six2*^{KI} scRNAseq from Combes and Jarmas, filtered similarly.

Supplementary Data 3. Markers of all scRNA-Seq clusters by sample age and genotype.

Supplementary Data 4. Annotations for barcode, cluster and cell type designation across all scRNA-Seq samples.

Supplementary Data 5. Clustering and top marker genes for integrated scRNA-Seq samples.

Supplementary Data 6. Clustering and marker genes for sex-based pseudo-bulk replicates.

Supplementary Data 7. Differential expression analysis between all cell populations for P0 *Six2*$^{TGC;Tsc1}$ vs. P0 *Six2*^{TGC} using cellHarmony

Supplementary Data 8. GO-Elite analysis of all nephron progenitor cells combined.

Supplementary Data 9. Translatome analysis of nephron progenitor cells.

Supplementary Data 10. Characterization of *Tmem59* mutant alleles.
